# Association between probiotic, prebiotic, and yogurt consumption and chronic kidney disease: The NHANES 2010–2020

**DOI:** 10.3389/fnut.2022.1058238

**Published:** 2022-12-23

**Authors:** Xiaoxian Liu, Wenyan Gao, Jie Yang, Genxiang Mao, Hong Lu, Wenmin Xing

**Affiliations:** ^1^Department of Nephrology, The Second Affiliated Hospital, Zhejiang University School of Medicine, Hangzhou, China; ^2^School of Pharmacy, Hangzhou Medical College, Hangzhou, China; ^3^Department of Nephrology, The Second Affiliated Hospital of Zhejiang Chinese Medical University, Hangzhou, China; ^4^Zhejiang Provincial Key Laboratory of Geriatrics, Department of Geriatrics, Zhejiang Hospital, Hangzhou, China; ^5^School of Pharmacy, Zhejiang Chinese Medical University, Hangzhou, China

**Keywords:** probiotics, prebiotic, yogurt, chronic kidney disease, NHANES findings

## Abstract

**Background:**

Previous studies suggested that gut dysbacteriosis may promote the occurrence of chronic kidney disease (CKD), and probiotic, prebiotic, or yogurt supplements may alleviate CKD progression. This study aims to examine the association between probiotic, prebiotic, or yogurt supplements and the risk of CKD using the data from NHANES.

**Methods:**

This study was designed to prospectively search data from the National Health and Nutrition Examination Survey (NHANES) (2011–2020). We examined dietary supplements and prescription medication labels to identify probiotic, or prebiotic product, and yogurt consumption during the dietary interview. The diagnosis of CKD was determined by the value of glomerular filtration rate (eGFR) and albumin creatinine ratio (ACR).

**Results:**

The study enrolled a total of 6,522 individuals. The prevalence of CKD was lower in the probiotic, prebiotic, or yogurt consumption group [age-adjusted odds ratio (OR): 0.77, 95% CI: 0.62–0.95, *P* = 0.02; multivariable-adjusted OR: 0.86, 95% CI: 0.69–1.07, *P* = 0.05]. Furthermore, 32% reduced risk was observed in the older group aged 55 years or older, and 32% reduced risk was also observed in the female population. Probiotic, or prebiotic, or yogurt supplements was associated a 12% reduction in moderate risk of CKD and an 11% reduction in very high risk of CKD.

**Conclusion:**

Our results suggest that probiotic, prebiotic, or yogurt supplements may contribute to the prevention of CKD and relieve its progression risk, especially in the female population and older population who were aged 55 years or older.

## 1 Introduction

Chronic kidney disease (CKD) is with a history of kidney injury lasting more than 3 months, which includes abnormalities of kidney structure or function induced by various factors (1). The clinical features of CKD are characterized by a reduced glomerular filtration rate (GFR) or increased urinary albumin excretion (albuminuria) (1). It is a slowly progressed onset of disease, which will develop into uremia without treatment. Currently, CKD is with a global prevalence of 11–13%, which will up to 34% in older than 70 years population following the prevalence increasing with age (2). Moreover, patients with CKD are probably liable for a high risk of mortality, accelerated cardiovascular disease (CVD), hypertension, obesity, and infections (3–6). A previous cohort study presented that patients with CKD induced a 83% higher of mortality than the all-cause mortality [hazard ratio (HR) = 1.83] (7). Despite the attention of scientists and clinicians, CKD care and treatment remains suboptimal; hence, novel drugs or treatment manner is urgently needed to explore.

Previous studies indicated that the imbalanced ecological system is closely related to multiple chronic diseases, such as CKD, diabetes mellitus, and cardiovascular disease (8). Evidence also indicated that the imbalance of gut microbiota could lead to chronic kidney disease. In turn, the deterioration of chronic kidney disease could also aggravate the imbalance of intestinal flora (9). Researchers proposed gut-kidney axis and CKD-colon axis to describe the interaction between kidney and intestine in 2011 (10) and 2015 (11). Then, probiotic, prebiotic supplements were expected to alleviate the progression of CKD by regulating the balance of intestinal flora. Currently, non-food probiotic and prebiotic supplements are becoming increasingly available in the United States (12). Probiotics, which consisting of active microorganisms, are beneficial to human health and regulate the balance of gut microbiota by regulating the systemic immune response (13). Current studies have shown that probiotic may have beneficial effect on patients with chronic kidney disease. Prebiotic consisted of non-digestive substrates to selectively stimulate the growth of healthy gut microbiota (14). In addition, yogurt contained at least 108 bacterial organisms per gram, which is the most common source of probiotic in dietary (15).

Although probiotic, prebiotic, or yogurt supplements may rescue the imbalanced gut microbiota in patients with CKD, there are still lack of extensive cross-sectional studies to evaluate the prevalence of CKD in probiotic, prebiotic, or yogurt supplements population. Therefore, this study aims to analysis the association of probiotic, prebiotic, or yogurt supplements with the prevalence of CKD as well as the risk of CKD progression by using the data from the National health and Nutrition Examination Survey (NHANSE) from 2011 to 2020.

## 2 Materials and methods

### 2.1 Study design and population

This study was designed as a cross-sectional study on patients with CKD using data from the NHANES from 2011 to 2020. This database was regularly updated and a nationally representative sample of about 5,000 persons was examined each year. This analysis included 6,522 patients with CKD (ages ≥ 18). Then we obtained participant information on demographic characters, health-related lifestyle, and complicated diseases, dietary habits (fiber, protein, etc.). Then these data were used to assess the association between probiotic, prebiotic, or yogurt supplements and the risk of CKD.

### 2.2 The diagnosis of CKD

The KDIGO 2021 guideline was used to define CKD. Briefly, the urinary albumin creatinine ratios (ACRs) and estimated glomerular filtration rate (eGFR) criteria were extracted from NHANES. ACRs were classified as less than 30 (A1), 30–300 (A2), or greater than 300 mg/g (A3). The eGFR was calculated using the Chronic Kidney Disease Epidemiology Collaboration (CKD-EPI) equation, which were classified into G1 (90 mL/min/1.73 m^2^), G2 (60–89 mL/min/1.73 m^2^), G3a (45–59 mL/min/1.73 m^2^), G3b (30–44 mL/min/1.73 m^2^), G4 (15–29 mL/min/1.73 m^2^), G5 (<15 mL/min/1.73 m^2^). And CKD patients were defined if they were with eGFR < 59 or ACRs > 30. Then, patients with CKD were further classified into three categories of prognostic risk for patient progression. In details, CKD patients were classified into moderate risk (G3a and A1 or G1–G2 and A2), high risk (G3b and A1, or G3a and A2, or G1–G2 and A3), and very high risk (G4–G5, G3b, and A2–A3 or G3a and A3) (16).

### 2.3 Assessment of probiotic, prebiotic, or yogurt supplements

From 2011 to 2020, in all NHANES years cycles, probiotic, or prebiotic supplements was collected. At present, no probiotic products are approved by the FDA. But lactulose is one prebiotic product regulated by FDA-regulated (17). Additionally, previous study had graphed a comprehensive list to identify products with prebiotics and probiotics (12), which was shown in Supplementary Table 1. And Food Frequency Questionnaire (FFQ) and Dietary Supplement Use 30-Day (DSQ) was used to evaluate probiotic, prebiotic and yogurt consumption (18) in participants whose age was ≥18. In details, text-mined for key phrase was used to identify prebiotics and probiotics, which included dietary supplement names and ingredients, and medication names and ingredients. Then, we classified the patient with CKD into two groups according to the consumption of probiotic, prebiotic or yogurt: no consumption of probiotic, or prebiotic, or yogurt, and consumption of prebiotic, or prebiotic, or yogurt, listed as in Table 1.

**TABLE 1:** Baseline population characteristics according to probiotic, prebiotic, and yogurt consumption in CKD patients (*n* = 6522).

	No probiotics, prebiotics, and yogurt consumption	Probiotics, prebiotics, or yogurt consumption	*P*-value
Participants [*n* (%)]	4,805 (73.67%)	1,717 (26.33%)	NA
**Socio-economic characteristics**			
Male [*n* (%)]	2,248 (75.02%)	623 (24.98%)	<0.01
Female [*n* (%)]	2,557 (63.80%)	1,094 (36.20%)	
Age (years), mean (SD)	52.00 (0.42)	52.93 (0.65)	0.15
**Ethnicity**			
White [*n* (%)]	2,166 (72.38%)	913 (80.47%)	<0.01
Black [*n* (%)]	1,015 (9.54%)	229 (4.75%)	
Mexican [*n* (%)]	553 (6.35)	137 (3.58%)	
Others [*n* (%)]	1,071 (11.73%)	438 (11.20%)	
**Healthy behavior factors**			
Smoke status			
Current smokers [*n* (%)]	868 (18.33%)	138 (7.79%)	<0.01
No current smokers [*n* (%)]	3,937 (81.67%)	1,579 (92.21)	
**Drinking**			
No drink user [*n* (%)]	652 (10.27%)	255 (10.92%)	<0.01
Former drink user [*n* (%)]	940 (15.77%)	259 (13.01%)	
Mild drink user [*n* (%)]	1,736 (39.27%)	749 (46.21%)	
Moderate drink user [*n* (%)]	707 (16.84%)	278 (19.25%)	
Heavy drink user [*n* (%)]	770 (17.84%)	176 (10.61%)	
**Physical activity level**			
Never [*n* (%)]	1,259 (22.78%)	315 (15.95%)	<0.01
Low [*n* (%)]	1,335 (28.02%)	543 (30.79%)	
Intermediate [*n* (%)]	1,244 (27.34%)	538 (34.85%)	
High [*n* (%)]	967 (21.86%)	321 (18.41%)	
**Dietary intake**			
Kcal/day (kcal), mean (SD)	2,046.15 (16.83)	2,058.32 (24.58)	0.68
Carbohydrates/day (g/100 kcal), mean (SD)	240.05 (1.87)	242.13 (2.99)	0.46
Protein/day (g/100 kcal), mean (SD)	80.11 (0.79)	85.21 (1.08)	0.01
Fiber/day (g/100 kcal), mean (SD)	17.18 (0.20)	20.34 (0.31)	<0.01
Total fat, mean (SD)	81.00 (0.81)	80.18 (1.14)	0.57
Vitamin C, mean (SD)	78.17 (1.98)	98.49 (2.57)	<0.01
**Chronic disease factors**			
**BMI (kg/m^2^), *n* (%)**			
BMI < 25	1,318 (26.64%)	566 (33.69%)	<0.01
BMI ≥ 25	3,487 (73.36%)	1,151 (66.31%)	
**Hypertension [*n* (%)]**			
No hypertension	2,344 (53.41%)	983 (61.86%)	<0.01
Hypertension	2,461 (46.59%)	734 (37.14%)	
**Diabetes [*n* (%)]**			
No diabetes	3,257 (71.98%)	1,283 (77.08%)	0.01
Diabetes	1,548 (28.02%)	434 (22.98%)	
**Chronic kidney disease**			
No CKD	3,779 (82.28%)	1,442 (85.15%)	0.03
CKD	1,026 (17.72%)	275 (14.85%)	

BMI, body mass index; NA, not applicable; CKD, chronic kidney disease; SD, standard deviation.

### 2.4 Definition of alcohol user, smoking, and physical activity

Alcohol user is classified as: no drink user, former drink user, mild drink user, moderate drink user, and heavy drink user based on a previous report (19). Smoking status is divided into former and current smokers. Current smokers were confirmed that to be currently smoking every day, or some days. Former smokers were confirmed to at least 100 cigarettes during their lifetime, but do not currently smoke (20). In addition, we classified patients with CKD into four categories of physical activity. The terms of physical activity included work activity, walking or bicycling activity, and recreational activity. Weekly metabolic equivalent (MET) minutes of physical activity was recorded and calculated into three quartiles, which were defined as never, low, intermediate, and high level of physical activity (20).

### 2.5 Statistical analysis

Continuous variables were presented as mean, standard deviation (SD), and categorical variables were presented as numbers or percentages. The demographic characteristics, including age, gender, race (white, black, Mexican, and other), and body mass index (BMI), were included. The dietary habit data had energy intake (kcal/day), carbohydrate intake (g/100 kcal), protein intake (g/100 kcal), fiber intake (g/100 kcal), total fat intake, and vitamin C intake. And we compared baseline characteristics using χ^2^ tests and one-way variance (ANOVA) analysis between probiotic, prebiotic, or yogurt consumption individuals with non-consumption individuals. The adjusted binary logistic regression models were performed to assess the association between probiotic, prebiotic and yogurt consumption and the prevalence of CKD, which was presented as OR and 95% confidence interval (95% CI). The potential risk factors for CKD were used in the multivariable-adjusted models, which were listed as follows: age, sex, smoking status, drinking, physical activity, and BMI (BMI < 25.0 kg/m^2^, BMI ≥ 25.0 kg/m^2^), hypertension (no hypertension and hypertension), diabetes (no diabetes and diabetes), and intake of energy, and total fat. Then, several adjusted models were conducted: model 1 (adjusted for age), model 2 (adjusted for age, sex, and race), model 3 (Model 2 plus smoke status, drinking status, and physical activity), model 4 (Model 2 plus BMI, hypertension, and diabetes). Further, to examine whether associations varied by population characteristics, we also performed the stratified analyses by age, sex, BMI, hypertension, and diabetes. R version 4.1.5 and nhanesR package was used for statistical analysis, a *P* < 0.05 was considered as statistically significant.

## 3 Results

### 3.1 Baseline characteristics according to probiotic, prebiotic, or yogurt consumption

Table 1 showed the baseline population characteristics for those who were intake probiotic, prebiotic, or yogurt. 4,805 (73.67%) individuals were in the non-probiotic, prebiotic, or yogurt consumption group, and 1,717 (26.33%) individuals were in the probiotic, prebiotic, or yogurt consumption group. Mean age of the two study population groups was 52.00 ± 0.42 and 52.93 ± 0.65 years, respectively. Participants in the probiotic, prebiotic, or yogurt consumption group were more likely to be female and White, compared to those in the no probiotic, prebiotic, or yogurt consumption group. In terms of lifestyle, individuals in the probiotic, prebiotic, or yogurt consumption group were less likely to smoke and heavy drink, were more physically active, and had less CKD, hypertension, obesity, or diabetes, compared to those in the no probiotic, prebiotic, or yogurt consumption group.

### 3.2 Modulation of CKD according to probiotic, prebiotic, or yogurt consumption

Associations between the probiotic, prebiotic, or yogurt consumption and the prevalence of CKD were shown in Table 2. Multivariable-adjusted models suggested a lower prevalence of CKD in the probiotic, prebiotic, or yogurt consumption individuals than that who were not. The age-adjusted OR (95% CI) was 0.77 (0.62–0.95) for the probiotic, prebiotic, or yogurt consumption group. The multivariable-adjusted OR (95% CI) was 0.79 (0.64–0.97) for the probiotic, prebiotic, or yogurt consumption group. Further adjustment of physical activity and diseases showed a marginally significant difference, with a decreasing trend.

**TABLE 2:** Multivariable-adjusted odds ratio (95% confidence intervals) of overall chronic kidney disease (CKD) by consumption of probiotic, prebiotic, or yogurt.

	No probiotics, prebiotics and yogurt consumption	Probiotics, or prebiotics, or yogurt consumption	*P*-value
CKD/Non-CKD	1,026/3,779	275/1,442	
Model 1	1.00 (ref)	0.77 (0.62–0.95)	0.02
Model 2	1.00 (ref)	0.79 (0.64, 0.97)	0.02
Model 3	1.00 (ref)	0.83 (0.75, 1.03)	0.04
Model 4	1.00 (ref)	0.86 (0.69, 1.07)	0.05

CKD, chronic kidney disease; Model 1: adjusted for age (years, continuous); model 2, adjusted for age, sex (male and female), race (White, Black, Mexican, and others); model 3, adjusted for all the factors in Model 2 plus smoke status (no current and current smoker), and drinking status (no drink user, former drink user, mild drink user, moderate drink user, and heavy drink user), physical activity (never, low, intermediate, and high); Model 4: adjusted for all the factors in Model 3 plus obesity (BMI < 25.0 kg/m^2^, BMI ≥ 25.0 kg/m^2^), hypertension (no hypertension and hypertension), diabetes (no diabetes and diabetes). *P*-value was tested from model including the ordinal variable of Probiotics, prebiotics, or yogurt consumption as a continuous term and using Wald test for it.

And then, we performed the stratified analysis based on population characteristics, including sex, race, obesity, hypertension, diabetes status, and HbA1c, to analyze the association between probiotic, prebiotic, or yogurt consumption and the prevalence of CKD, shown as in Table 3. Race, BMI, and hypertension were not likely to significantly modify the association; however, sex and diabetes status significantly modified the association between probiotic, prebiotic, or yogurt consumption and the prevalence of CKD (*P* < 0.05). Specifically, in the group of female population, the multivariable OR (95% CI) was 0.68 (0.51–0.90) for the probiotic, prebiotic, or yogurt consumption group. Moreover, when the CKD patients were stratified by the age 55 years cut-off reported by previous study (21), we found that the multivariable OR (95% CI) was 0.68 (0.52–0.88) for the probiotic, or prebiotic or yogurt consumption group compared to the non-consumption group (*P* < 0.01) in older population (age ≥ 55 years). In contrast, no significant associations were observed in adults whose age < 55 years.

**TABLE 3:** Multivariable-adjusted odds ratio (95% confidence intervals) of overall chronic kidney disease (CKD) by use of probiotic, prebiotic, or yogurt according to population characteristics.

Stratification factors	CKD cases/non- CKD cases	No probiotics, prebiotics, and yogurt consumption	Probiotics, prebiotics, or yogurt consumption	*P*-value
**Age**				
<55 years	911/2,410	1.00 (ref)	1.06 (0.65, 1.72)	0.81
≥55 years	806/2,395	1.00 (ref)	0.68 (0.52, 0.88)	<0.01
**Sex**				
Male	623/2,248	1.00 (ref)	0.91 (0.56, 1.49)	0.71
Female	1,094/2,557	1.00 (ref)	0.68 (0.51, 0.90)	0.01
**Ethnicity**				
White	913/2,166	1.00 (ref)	0.81 (0.63, 1.05)	0.11
Black	2,229/1,015	1.00 (ref)	0.66 (0.39, 1.12)	0.12
Mexican	137/553	1.00 (ref)	0.71 (0.36, 1.37)	0.29
**BMI**				
BMI < 25 kg/m^2^	566/1,318	1.00 (ref)	0.79 (0.55, 1.13)	0.19
BMI ≥ 25 kg/m^2^	1,151/3,487	1.00 (ref)	0.79 (0.62, 1.00)	0.05
**Hypertension**				
Normal	983/2,344	1.00 (ref)	0.86 (0.59, 1.26)	0.44
Hypertension	734/2,461	1.00 (ref)	0.80 (0.63, 1.03)	0.08
**Diabetes**				
Normal	1,283/3,257	1.00 (ref)	0.88 (0.67, 1.16)	0.35
Diabetes	434/1,548	1.00 (ref)	0.70 (0.50, 0.98)	0.01

CKD, chronic kidney disease. Adjusted for the same variables included in the multivariable model 4 of Table 2.

### 3.3 Modulation of risk of CKD progression according to probiotic, prebiotic, or yogurt consumption

The age-adjusted and multivariable-adjusted models were used to evaluate the association between the risk of CKD progression and probiotic, prebiotic, or yogurt consumption, as shown in Table 4. Probiotic, prebiotic, or yogurt supplements showed a 14% reduction risk for CKD cases with moderate risk in the age-adjusted model with a marginally statistical difference, whereas it just showed a 12% reduction risk in multivariable-adjusted model without significant difference. It showed an 11% reduction risk for CKD cases with very high risk in the age-adjusted model with a marginally significant difference compared probiotic, prebiotic, or yogurt supplements with those who didn’t supplement. However, there was just a 3% reduction in CKD cases with high risk of progression in the age-adjusted model with a marginally significant difference.

**TABLE 4:** Multivariable-adjusted odds ratio (95% confidence intervals) of chronic kidney disease (CKD) subtypes defined by its risk of progression by use of nutrition labels.

Stratification factors	No probiotics, prebiotics, and yogurt consumption	Probiotics, prebiotics, or yogurt consumption	*P*-value
**CKD cases with moderate risk of progression**			
Cases/Non-cases	655/3,779	187/1,442	
Age-adjusted	1.00 (ref)	0.86 (0.66, 1.13)	0.05
Multivariable-adjusted	1.00 (ref)	0.88 (0.66, 1.16)	0.06
**CKD cases with high risk of progression**			
Cases/Non-cases	207/3,779	64/1,442	
Age-adjusted	1.00 (ref)	0.97 (0.66, 1.44)	0.05
Multivariable-adjusted	1.00 (ref)	0.98 (0.70, 1.39)	0.07
**CKD cases with very high risk of progression**			
Cases/Non-cases	164/3,779	24/1,442	
Age-adjusted	1.00 (ref)	0.89 (0.51, 1.54)	0.05
Multivariable-adjusted	1.00 (ref)	0.89 (0.51, 1.53)	0.08

CKD, chronic kidney disease. Adjusted for the same variables included in the multivariable model 4 of Table 2.

## Discussion

In the present cross-sectional study of nationally representative data in the USA, we found that probiotic, prebiotic, or yogurt supplements were associated with a 23% lower prevalence of CKD in adult individuals. We also observed a significantly lower risk of CKD progression with probiotic, prebiotic, or yogurt consuming, which was more pronounced in CKD cases with a moderate and very high risk of progression than CKD cases with high risk of progression. These associations are still obtained after adjusting for potential confounders, such as age, sex, and so on. Furthermore, sex, hypertension, and diabetes status, significantly modified the association between probiotic, prebiotic, or yogurt supplements and the prevalence of CKD. The present study is the first comprehensive, large epidemiologic analysis to investigate the association between probiotic, prebiotic, or yogurt supplements and the prevalence of CKD and risk of CKD progression. Our results indicated that gut microbiota modification might potentially contribute to the prevention of CKD overall and delay its early progression.

Previous evidence had shown the benefits of probiotic and prebiotic supplements in CKD patients. For instance, Yacoub et al. analyzed the association of probiotic alone (1999–2012) and yogurt/probiotic (2003–2006) use with albuminuria and eGFR from NHANES data. It found that participants frequently intake of yogurt/probiotics had less urinary albumin excretion compared to these who infrequent intake. Moreover, probiotic intake alone had a lower albuminuria compared to non-intake (OR = 0.59). Whereas, there was no association was found between yogurt/probiotics intake and estimated glomerular filtration rate (eGFR) decreasing (18). Other clinical studies found that there was a decrease in the serum urea concentrations in CKD patients with Stages 3 and 4 after using 16 billion CFU/day of *Lactobacillus* for 2 months (22). The blood urea nitrogen levels were significantly decreased by using 90 billion CFU/day of probiotic formulation for 6 months in 29 CKD patients (23). However, it showed no effect on uremic toxins in CKD patients with hemodialysis after 2 months of supplements with a 180 billion CFU/day dose of the probiotic formulation (24). Essentially, prebiotic or symbiotic supplements can modulate the imbalanced gut microbiota in CKD patients. Simultaneously, it can improve the integrity of the intestinal epithelial barrier, decrease uremic toxins production and attenuate local and systemic inflammation. In addition, metabolites derived from gut microbiota also played a profound role in maintaining of gut homeostasis to benefit host health through the fermentation of amino acids and dietary fiber, generation of vitamins and neurotransmitters, and modification of bile acids (25). For instance, the short-chain fatty acids (SCFAs) derived from multiple bacteria can positively regulate the effects on immune inflammation and protect against acute tubular injury in acute kidney injury (AKI) (26). Indeed, probiotic or prebiotic consumption can inverse the expansion of harmful gut microorganisms producing excessive amounts of uremic toxins and attenuating the development of CKD (27, 28).

In addition, one previous meta-analysis showed that probiotic or synbiotic supplementation significantly improved the glutathione (GSH) level to contribute to the host oxidative stress homeostasis (29). Moreover, microbial supplements can reduce the level of pro-inflammatory biomarker (CRP), improve the oxidative unbalance among pro-oxidant factors and anti-oxidant enzymes (malondialdehyde, GSH, and TAC), and facilitate the lipid profile (cholesterol, high-density lipoprotein cholesterol, and low-density lipoprotein cholesterol) in CKD patients (30). In addition to microbial medications consumption, health lifestyle modification would be an effective way to reshape the imbalanced gut microbiota into a healthier phenotype. For example, it could improve the abundance of *Prevotella*, *Lactobacilli*, and *Bifidobacteria* and inhibit the growth of *Bacteroides*, *Enterobacteria*, and *Clostridia* through an enriched fruit or vegetable diet (31). In addition, an enriched amylose diet could also reduce inflammation and renal fibrosis, retard the progression of kidney disease, by improving gut microbial dysbiosis in rats with CKD (32, 33). Moreover, fiber enriched diet has been confirmed to inverse the decline in glomerular filtration rate, and reduce inflammation and mortality in CKD patients (34, 35).

In the present study, hypertension, and diabetes status may significantly affect the association between the use of probiotic, prebiotic, or yogurt and the prevalence of CKD. Indeed, hypertension and CKD influence promotes each other, which could impair epithelial barrier structure and function and reduce tissue perfusion (31, 36). Consequently, the imbalance of gut microbiota was exacerbated in CKD patients. In detail, the increase of *Escherichia* and the reduction of *Lactobacilli* and *Lachnospiraceae* could increase bacterial adhesiveness and virulence (37). Another factor affecting the association of between use of probiotic, prebiotic, or yogurt and the prevalence of CKD was the complication of diabetes. Previous reports showed that gut dysbiosis directly promoted the development of both type 1 and type 2 diabetes (38). As a result, the profound role of gut dysbiosis may influence the efficiency of microbial supplements in CKD patients with hypertension or diabetes complication.

In addition, we observed a lower of 14 and 11% for moderate and very high risk of CKD progression, respectively, compared with prebiotic, probiotic, or yogurt consumption individuals and those who didn’t consume. However, there is just a slightly reduction for high risk of CKD progression. Furthermore, the significance of both high and very high risk of CKD progression was just marginal. Possibly, individuals with “high” and “very high” risk of CKD progression tend to be involved in other complications, such as hypertension, and diabetes (39). On the other hand, individuals with “high” and “very high” risk of CKD progression often restrict their diet according to medication orders, such as low intake fiber.

Therefore, the present study had several limitations for interpretation of the results. First, the NHANES assessed the use of probiotic or prebiotic based on self-reported information and manufacturers’ label information; i.e., the probiotic or prebiotic is mainly source of non-food (12). Second, we also defined probiotic supplements as yogurt consumption. And the population was classified according to whether or not they use probiotic, prebiotic, or yogurt supplements. But the duration, and quantity of supplemental were not taken into considered, which may affect the association between probiotic or prebiotic consumption and the prevalence of CKD. In addition, fatty acids, phenolics, or phytochemicals were not included as prebiotic for lack of scientific consensus (12). Third, obesity and diabetes complications might influence the benefit of prebiotic, probiotic and yogurt supplements, the results interpretation should be cautious for CKD patients complicated with diabetes and obesity. Fourth, there were likely absence of medications information for CKD patients in NHANES, the results needed to be cautiously interpreted.

## Conclusion

This is the first nationally representative cross-sectional study based on the USA population to analyze the beneficial association of probiotic, prebiotic supplements, yogurt consumption with the prevalence of CKD. The use of probiotic, prebiotic supplements, or yogurt consumption was found to be associated with a reduction prevalence of CKD, and it also related to the decrease risk of CKD cases with a moderate and very high risk of progression. These results further provide novel insights on probiotic or prebiotic as an effective tool in the prevention and management of CKD. But it should concern the individual’s sex, age, particular the complications, which could influence the effect of the probiotic, prebiotic and yogurt supplements. Future researches need to pay more attention on understanding the gut microbiota in the development of CKD and identifying individuals who benefit the most from selective modulation of microbiota.

## Data availability statement

The original contributions presented in this study are included in the article/Supplementary material, further inquiries can be directed to the corresponding authors.

## Author contributions

WX and XL were responsible for the design. WX, GM, and HL were responsible for writing of the work. WG and JY performed the data extraction. WG and WX were responsible for the data analysis. All authors reviewed the draft and approved the final manuscript.
