# Supplementary material for: Association between probiotic, prebiotic, and yogurt consumption and chronic kidney disease: The NHANES 2010–2020
Source: Front Nutr. 2022 Dec 23;9:1058238. doi: 10.3389/fnut.2022.1058238 (PMC9822650; doi:10.3389/fnut.2022.1058238)
Supplement: Supplementary file 1 [file Table_1.DOCX]

**­­­Supplementary Table 1. Search terms used to identify dietary supplements or prescription drugs that were either labeled with or contained ingredients labeled as prebiotics, probiotics in NHANES[1].**

| Prebiotic search terms[2] | “ACACIA GUM”, “CHICOR”, “GLUCAN”, “GUM ARABIC, “INULIN”, “LACTULOSE”, “OLIGOFRUC”, “OLIGOSAC”, “POLYDEXTROSE”, “PREBIOTIC”, “PRE-BIOTIC”, “PRE BIOTIC”, “PSYLLIUM”, “RESISTANT STARCH”, “WHEAT DEXTRIN” |
| --- | --- |
| Probiotic search terms[3, 4] | “ACIDOPHILUS”, “ANIMALIS”, “BACILLUS”, “BACILLI”, “BIFIDOBACTERI”, “BIFIDUM”, “BOULARDII”, “BREVE”, “BREVIS”, “BUCHNERI”, “BULGARICUS”, “BUTYRICUM”, “CASEI”, “CAUCASICUS”, “CEREVISIAE”, “CLAUSII”, “CLOSTRIDI”, “COAGULANS”, “CORYNIFORMIS”, “CRISPATUS”, “DELBRUECKII”, “ESCHERICH”, “E. COLI”, “ECOLI”, “E COLI”, “ENTEROCOCCUS”, “FAECALIS”, “FAECIUM”, “FERMENTUM”, “FLORENTINUS”, “GASSERI”, “HELVETICUS”, “INFANTIS”, “JOHNSONII”, “LACTIS”, “LACTIC ACID BACTERIA”, “LACTOBACILL”, “LACTOCOCCUS”, “LEICHMANNII”, “LEUCONOSTOC”, “LICHENIFORMIS”, “LONGUM”, “MESENTERIC”, “MITIS”, “NISSLE”, “OLIGONITROPHILUS”, “ORALIS”, “PARACASEI”, “PEDIOCOCCUS”, “PLANTARUM”, “PROBIOTIC”, “PRO-BIOTIC”, “PRO BIOTIC”, “PROPIONIBACTERI”, “RATTUS”, “REUTERI”, “RHAMNOSUS”, SACCHAROMYC”, “SALIVARIUS”, “SANGUIS”, “STEAROTHERMOPHILUS”, “STREPTOCOCCUS”, “SUBTILIS”, “THERMOPHILUS” |

NOTE. Variations of terms (such as “BACTERIA” and “BACTERIUM”) were tested before curating this final list to ensure high sensitivity and specificity of the search terms.

**references**

1. O'Connor LE, Gahche JJ, Herrick KA, Davis CD, Potischman N, Vargas AJ: **Nonfood Prebiotic, Probiotic, and Synbiotic Use Has Increased in US Adults and Children From 1999 to 2018**. *Gastroenterology* 2021, **161**(2):476-486.e473.

2. Pineiro M, Asp NG, Reid G, Macfarlane S, Morelli L, Brunser O, Tuohy K: **FAO Technical meeting on prebiotics**. *J Clin Gastroenterol* 2008, **42 Suppl 3 Pt 2**:S156-159.

3. Hempel S, Newberry S, Ruelaz A, Wang Z, Miles JN, Suttorp MJ, Johnsen B, Shanman R, Slusser W, Fu N *et al*: **Safety of probiotics used to reduce risk and prevent or treat disease**. *Evid Rep Technol Assess (Full Rep)* 2011(200):1-645.

4. Su GL, Ko CW, Bercik P, Falck-Ytter Y, Sultan S, Weizman AV, Morgan RL: **AGA Clinical Practice Guidelines on the Role of Probiotics in the Management of Gastrointestinal Disorders**. *Gastroenterology* 2020, **159**(2):697-705.
